# Mental health status and influencing factors of retired teachers

**DOI:** 10.3389/fpubh.2024.1358285

**Published:** 2024-06-05

**Authors:** Pingyun Luo, Yuanzhen Liu, Xianjiang Pan, Liyun Huang

**Affiliations:** ^1^The Faculty of Education, Southwest University, Chongqing, China; ^2^The Academic Affairs Office of Qiannan Preschool Education College for Nationalities, Guiding, Guizhou, China; ^3^The Office of Guangzhou Children's Palace, Guangzhou, Guangdong, China

**Keywords:** retired teachers, psychological health, influence factors, obsessive-compulsive disorder, interpersonal sensitivity

## Abstract

**Introduction:**

The wellbeing of retired teachers is often easily overlooked. This study aims to explore the mental health status and influencing factors of retired teachers.

**Method:**

From October to December 2022, a convenient sampling survey was conducted on retired teachers using the Symptom Checklist 90 (SCL-90), mainly using the χ^2^-test and logistic regression analysis.

**Results:**

A sampling survey was conducted on 353 retired teachers, with an overall positive detection rate of 16.1%. The five factors with the highest positive detection rate were found to be obsessive-compulsive disorder (30.3%), interpersonal sensitivity (21.5%), paranoia (20.1%), anxiety (19.3%), and others (19.3%). The detection rates for the five factors, namely psychosis, depression, hostility, terror, and somatization, are all below 19%. The data on sex (χ^2^ = 4.626, *P* = 0.043), professional title (χ^2^ = 17.670, *P* = 0.003), income (χ^2^ = 9.960, *P* = 0.041), life satisfaction (χ^2^ = 27.348, *P* = 0.000), family relationships (χ^2^ = 51.451, *P* = 0.000), and physical health status (χ^2^ = 50.361, *P* = 0.000) show that the difference in mental health among retired teachers is statistically significant. The multivariate binary logistic regression analysis revealed that family relationships, life satisfaction, and physical health were important factors leading to mental health problems among retired teachers.

**Discussion:**

Retired teachers should cultivate a wide range of interests and hobbies, engage in regular physical exercise, develop healthy living habits, foster a positive family atmosphere, establish harmonious family relationships, promote community cultural construction, strengthen psychological intervention, and prevent psychological diseases.

## 1 Introduction

In February 2023, the National Bureau of Statistics released the 2022 National Economic and Social Development Statistical Bulletin. The report showed that at the end of 2022, the population of people aged 60 and above in China was 280 million (1). Among them, retired teachers form an important component of the older adult population in China. They not only share the common psychological characteristics of the Chinese older adult but also possess unique characteristics specific to their group. In recent years, retired teachers have mental health problems such as anxiety, paranoia, depression, fear, and sadness, making it challenging for them to adapt to their new roles (2). Meng and others suggest that the transition into retirement has led to 85% of older adult people experiencing varying degrees of psychological problems (3). Wu pointed out that the older adult have mental health problems mainly due to factors such as retirement, insufficient care from children, health issues, economic and life challenges, and the loss of loved ones. When old people's mental health is neglected, it will lead to some mental health problems, including feelings of loss, fear, loneliness, depression, dementia, and other psychological problems (4). Huang et al. demonstrated that the mental health status of the older adult is significantly affected by the level of their self-concept, occupation, and education level (5).

However, by searching China National Knowledge Infrastructure (CNKI) with the keywords “retired teachers” and “mental health,” it was found that research on retired teachers mainly focuses on “life satisfaction” and “health management models,” while the research on mental health of retired teachers is very limited. This study investigates the mental health status of retired teachers through a survey questionnaire, aiming to understand the mental health status of retired teachers, explore the main factors affecting their mental health, and provide reference for the development of psychological health intervention measures for retired teachers.

## 2 Materials and methods

### 2.1 Respondents

A total of 410 questionnaires were distributed across various locations including Qianling Mountain, Guiding Jinshan Park, Guiding Moon Mountain Park, Beibei Sports Park, Beibei Riverside Trail, Qiannan Prefecture University for the older adult. Out of these, 390 questionnaires were recovered, of which 353 were deemed valid, with an effective recovery rate of 86.1%.

### 2.2 Survey method

The study used a stratified sampling method. We first sampled cities according to their geographical distribution. Then, we obtained information on the distribution areas of the older adult according to the data from the National Statistical Office's 2020 sample survey. Finally, we identified areas for sampling. A questionnaire survey method was used for on-site investigation. The survey personnel included trained and qualified college students who explained the meaning of the question to the respondents in a neutral, non-suggestive, and unbiased manner. The questionnaire was filled out with the help of survey personnel due to factors such as respondents' vision and physical health. The surveyed individuals were all voluntary participants, except for those with intellectual disabilities or who could not express their opinions clearly.

### 2.3 Survey tool

The survey questionnaire consists of two parts: (2) Basic information of retired teachers including ethnicity, sex, age, teaching experience, educational background, professional title, marriage, income, life satisfaction, family relationships, physical health, etc. (3) The survey used the SCL-90 scale (Symptom Checklist). The SCL-90 consists of 90 items and 10 symptom factors, covering a wide range of psychiatric symptoms, which include feelings, emotions, thought processes, states of consciousness, behaviors, lifestyle habits, interpersonal relationships, dietary habits, and sleep patterns. Each project is assigned a rating between 1 and 5 points (6). The validity coefficients for each factor in the scale range from 0.77 to 0.90, with an internal consistency reliability of 0.98, indicating strong reliability and validity.

### 2.4 Statistical method

This study uses SPSS24.0 software to perform χ^2^ tests and logistic regression analysis.

### 2.5 Research hypotheses

First, the mental health status of retired teachers is generally low; second, there is a significant difference between the mental health status and that of other groups of retired senior citizens; thirdly, the mental health of retired teachers is affected by factors such as family relationships, life satisfaction, and physical health.

### 2.6 Research objectives

Through researching the mental health status of retired teachers, this study seeks to analyze the current situation of retired teachers' mental health, identify the influencing factors of retired teachers' mental health, and put forward measures to improve the mental health of retired teachers from various aspects based on the influencing factors.

## 3 Results

### 3.1 Basic information of retired teachers

Out of 353 retired teachers, 176 were of Han nationality (49.9%); 32 were male individuals (9.1%); 340 people (96.3%) had a college degree; 346 individuals (98%) were without spouses (including unmarried, divorced, or separated); 77.3% of retired teachers had a monthly income of <3,000 yuan; 36.8% of the participants were very satisfied with their current life; 39.4% had harmonious family relationships; and 39.1% of them had good physical health.

### 3.2 SCL-90 positive detection status

The overall positive detection rate is found to be 16.1%. The five factors with the highest positive detection rate include obsessive-compulsive disorder (30.3%), interpersonal sensitivity (21.5%), paranoia (20.1%), anxiety (19.3%), and others (19.3%). The detection rates of the five factors, namely psychosis, depression, hostility, terror, and somatization, were all below 19%. See Table 1.

**Table 1:** Symptom checklist 90 (SCL-90) detection status (n = 353).

**Variable quantity**	**Frequency**	**Percentage (%)**	**Sorting of positive rates**
Somatization			10
Negative	309	87.5	
Positive	44	12.5	
Obsessive-compulsive disorder			1
Negative	246	69.7	
Positive	107	30.3	
Interpersonal Sensitivity			2
Negative	277	78.5	
Positive,	76	21.5	
Paranoia			7
Negative	291	82.4	
Positive	62	17.6	
Anxiety			4
Negative	285	80.7	
Positive	68	19.3	
Hostile			8
Negative	295	83.6	
Positive	58	16.4	
Terror			9
Negative	300	85.0	
Positive	53	15.0	
Paranoia			3
Negative	282	79.9	
Positive	71	20.1	
Psychiatric			6
Negative	289	81.9	
Positive	64	18.1	
Other			5
Negative	285	80.7	
Positive	68	19.3	
Total detection			
Negative	296	83.9	
Positive	57	16.1	

### 3.3 Single-factor analysis of the overall physical examination rate of retired teachers in SCL-90

The results of single-factor analysis of sex (χ^2^ = 4.626, *P* = 0.043), professional title (χ^2^ = 17.670, *P* = 0.003), income (χ^2^ = 9.960, *P* = 0.041), life satisfaction (χ^2^ = 27.348, *P* = 0.000), family relationships (χ^2^ = 51.451, *P* = 0.000), and physical health status (χ^2^ = 50.361 *P* = 0.000) reveal that the difference in mental health status among retired teachers is statistically significant. See Table 2.

**Table 2:** Single-factor analysis of the total detection rate of SCL-90 (*n* = 353).

**Factor**	**Total detection**		**χ^2^**	***P*-value**
	**Negative**	**Positive**		
Nation			0.169	0.773
Han nationality	149	27		
Minority nationality	147	30		
Sex			4.626	0.043
Male	32	1		
Female	264	56		
Education			2.013	0.633
Middle school and below	2	0		
Junior college	286	54		
Bachelor degree	7	3		
Master degree	1	0		
Technical title			17.670	0.003
Primary and Secondary School level 3	203	49		
Primary and Secondary School Level 2	65	1		
Primary and Secondary School level 1	18	3		
Deputy Senior titles	3	3		
Senior titles	7	1		
Income			9.960	0.041
≦4,000¥	134	33		
4,001–5,000¥	89	17		
5,001–6,000¥	62	3		
6,001–7,000¥	6	3		
>7,000¥	5	1		
Life satisfaction			27.348	0.000
Very satisfied	117	13		
Quite satisfied	121	22		
Generally satisfied	56	16		
Not very satisfied	2	4		
Very dissatisfied	0	2		
Family relationships			51.451	0.000
Very good	130	9		
Quite good	97	16		
Commonly	67	23		
Relatively bad	2	7		
Very bad	0	2		
Physical health			50.361	0.000
Very good	129	9		
Quite good	99	17		
Commonly	66	22		
Relatively poor	2	7		
Very poor	0	2		

### 3.4 Mental health influencing factors of retired teachers

Based on single-factor analysis, we performed multivariate binary logistic regression analysis, and the results revealed that family relationships, life satisfaction, and physical health were significant factors leading to mental health problems among retired teachers (see Table 3).

**Table 3:** Mental health logistic regression analysis of retired teachers.

	**B**	**S.E**.	**Wald**	***P* value**	**OR**	**95% CI**	
						**Lower**	**Upper**
Nationality	−0.233	0.339	0.473	0.492	0.792	0.407	1.540
Marital status	0.275	0.536	0.265	0.607	1.317	0.461	3.763
Technical title	0.261	0.220	1.409	0.235	1.298	0.844	1.998
Family relationships	0.627	0.223	7.880	0.005	1.872	1.208	2.900
Life satisfaction	0.498	0.223	4.973	0.026	1.645	1.062	2.548
Physical health	0.844	0.226	13.923	0.000	2.326	1.493	3.625
Education	0.222	0.735	0.091	0.763	1.248	0.296	5.271
Income	0.030	0.208	0.021	0.886	1.030	0.686	1.547

## 4 Discussion

This study demonstrated that the overall positive detection rate of mental health problems among retired teachers was 16.1%, which is approximately similar to the findings of Wang and Liu (7) regarding the mental health status of the older adult (20.21%). Among the 10 factors tested, the factor with the highest positive detection rate was obsessive-compulsive disorder, which is closely consistent with the high detection rate of obsessive-compulsive disorder found in the study by Wang and Chen (8). Compulsive symptoms seriously endanger the mental health of the older adult, thereby affecting their life satisfaction and physical health (9). The single-factor analysis revealed that the positive rate of mental health status among female retired teachers was higher than that of men. However, this finding was not confirmed through logistic regression analysis. While professional title, income, life satisfaction, family relationships, and physical health status all have an impact on the mental health of retired teachers, logistic regression analysis reveals that only family relationships, life satisfaction, and physical health status have an impact on the positive detection rate of retired teachers. Family relationships affect the positive detection rate of retired teachers. Harmonious family relationships are beneficial for retired teachers to regulate and channel their negative emotions, especially through the care received from their children or spouses. This directly affects the severity of symptoms such as compulsion, interpersonal sensitivity, anxiety, and paranoia, which is beneficial for reducing the occurrence of adverse psychological symptoms. Life satisfaction impacts the positive detection rate of retired teachers, which is consistent with the findings of Dai et al. (10) on the mental health of older adult university students. To a certain extent, life satisfaction can significantly influence the emotional state of retired teachers. When life satisfaction is high, it can enhance their joy and overall happiness. On the contrary, low life satisfaction can lead to anxiety and potentially have negative effects on their mental health. Numerous studies (11–13) have shown that physical health is an important factor affecting the mental health of retired teachers, which is consistent with the conclusions of this study. As retired teachers grow older, their bodily organs gradually decline and lead to physical discomfort. Chronic diseases can trigger adverse psychological symptoms, such as depression, obsessive-compulsive disorder, and anxiety, and even lead to mental illness, which indicates the importance of physical health for the mental health of retired teachers.

### 4.1 Suggestions

First, retired teachers should cultivate a wide range of interests and hobbies and develop healthy living habits. Retired teachers must go through a period of adaptation from their professional lives to family life. During this period, they can cultivate hobbies, such as gardening, feeding small animals, cooking, playing musical instruments, and singing, among others. In addition, they can also develop healthy dietary habits suited to their individual health needs, especially by including fresh fruits and vegetables in each meal. Cultivating healthy living habits is beneficial for retired teachers to smoothly and healthily transition through the adaptation period.

Second, creating a positive family atmosphere and establishing harmonious family relationships can have an impact on the mental health status of retired teachers. The report from the 20^th^ National Congress of the Communist Party of China emphasizes the importance of “strengthening the construction of family education and family conduct.” Regarding the psychological health of retired teachers, it is necessary to thoroughly study and implement the principles of the 20^th^ National Congress of the Communist Party of China along with General Secretary Xi Jinping's significant statements on family tutoring and family conduct. By continuously upholding and strengthening the construction of family ethics through family tutoring, we can create positive family and social environments. In this process, the government, society, and families should work closely to promote the mental health status of retired teachers. As a retired teacher, it is even more important to strictly demand oneself and pay attention to the methods and arts of creating a family atmosphere in the construction of family style through personal guidance.

Third, research has found that physical health can significantly improve mental health. Retired teachers should prioritize physical exercise, especially morning and evening routines, in their daily lives. This practice not only helps in maintaining physical fitness but also provides opportunities to connect with fellow retirees during these activities. Engaging in such exercises can relieve inner stress and offer a platform for sharing experiences and receiving psychological counseling from peers.

Fourth, retired teachers should be involved in promoting community cultural construction and improving life satisfaction. Neighborhoods and communities should incorporate cultural construction into their annual work priorities, with the goal of improving the satisfaction of community people's lives. Retired teachers, as valuable sources of knowledge resources for neighborhoods and communities, must fully and reasonably utilize their “surplus energy.” In the process of participating in cultural construction activities, these retired teachers not only recognize their own value but also derive high-quality life satisfaction from the achievements of cultural construction.

Finally, strengthening psychological intervention to prevent psychological diseases is crucial. Every year, communities can organize psychologists to conduct free mental health surveys for retired teachers. Timely follow-up and regular intervention should be carried out for screened positive patients. Volunteers from universities and hospitals can be invited to the community to carry out mental health lectures, group counseling, group psychotherapy, and other activities. Neighborhoods and communities can also establish psychological counseling rooms and hire social volunteers or retired teachers with psychological backgrounds as psychological counselors.

There are still some limitations in this study. First, the sample size of the survey is small, and the representativeness of the sample is slightly less convincing. Second, the sampling method should be more precise by increasing the sample size and coverage of the survey in the future. Finally, it is also important to note that sampling bias is unavoidable, which may impact the recommendations for improving the mental health of retired teachers.

## Data availability statement

The original contributions presented in the study are included in the article/supplementary material, further inquiries can be directed to the corresponding authors.

## Ethics statement

Ethical approval was not required for the study involving human participants in accordance with the local legislation and institutional requirements. Written informed consent to participate in this study was not required from the participants in accordance with the national legislation and the institutional requirements.

## Author contributions

PL: Conceptualization, Data curation, Formal analysis, Funding acquisition, Investigation, Methodology, Project administration, Resources, Software, Supervision, Validation, Visualization, Writing – original draft, Writing – review & editing. YL: Investigation, Writing – review & editing, Methodology. XP: Investigation, Writing – review & editing, Formal analysis. LH: Investigation, Writing – review & editing.
